# Dichlorido(2,3-di-2-pyridyl­pyrazine-κ^2^ *N* ^1^,*N* ^2^)platinum(II)

**DOI:** 10.1107/S1600536811038906

**Published:** 2011-09-30

**Authors:** Kwang Ha

**Affiliations:** a School of Applied Chemical Engineering, The Research Institute of Catalysis, Chonnam National University, Gwangju 500-757, Republic of Korea

## Abstract

The Pt^II^ ion in the title complex, [PtCl_2_(C_14_H_10_N_4_)], is four-coordinated in a distorted square-planar environment by two N atoms of a chelating 2,3-di-2-pyridyl­pyrazine ligand and two chloride anions. The pyridyl ring coordinated to the Pt^II^ atom is inclined slightly to its carrier pyrazine ring [dihedral angle = 13.5 (1)°], whereas the uncoordinated pyridyl ring is inclined considerably to the pyrazine ring [dihedral angle = 54.3 (2)°]. The dihedral angle between the two pyridyl rings is 59.2 (2)°. In the crystal, the complexes are assembled through inter­molecular C—H⋯N and C—H⋯Cl hydrogen bonds, forming a three-dimensional network. Intra­molecular C—H⋯N and C—H⋯Cl hydrogen bonds are also present.

## Related literature

For the synthesis and crystal structure of [PtBr_2_(C_14_H_10_N_4_)], see: Ha (2011 ▶).
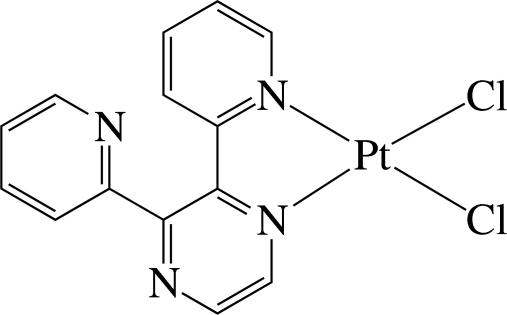


## Experimental

### 

#### Crystal data

[PtCl_2_(C_14_H_10_N_4_)]
*M* *_r_* = 500.25
Monoclinic, 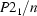

*a* = 8.894 (5) Å
*b* = 9.711 (5) Å
*c* = 16.461 (9) Å
β = 94.429 (11)°
*V* = 1417.5 (13) Å^3^
*Z* = 4
Mo *K*α radiation
μ = 10.27 mm^−1^
*T* = 200 K
0.28 × 0.15 × 0.11 mm

#### Data collection

Bruker SMART 1000 CCD diffractometer
Absorption correction: multi-scan (*SADABS*; Bruker, 2000 ▶) *T*_min_ = 0.638, *T*_max_ = 1.000
9749 measured reflections
3370 independent reflections
2756 reflections with *I* > 2σ(*I*)
*R*_int_ = 0.029

#### Refinement

*R*[*F* ^2^ > 2σ(*F* ^2^)] = 0.024
*wR*(*F* ^2^) = 0.056
*S* = 1.02
3370 reflections
190 parameters
H-atom parameters constrained
Δρ_max_ = 1.83 e Å^−3^
Δρ_min_ = −0.83 e Å^−3^

### 

Data collection: *SMART* (Bruker, 2000 ▶); cell refinement: *SAINT* (Bruker, 2000 ▶); data reduction: *SAINT*; program(s) used to solve structure: *SHELXS97* (Sheldrick, 2008 ▶); program(s) used to refine structure: *SHELXL97* (Sheldrick, 2008 ▶); molecular graphics: *ORTEP-3* (Farrugia, 1997 ▶) and *PLATON* (Spek, 2009 ▶); software used to prepare material for publication: *SHELXL97*.

## Supplementary Material

Crystal structure: contains datablock(s) I. DOI: 10.1107/S1600536811038906/tk2794sup1.cif

Structure factors: contains datablock(s) I. DOI: 10.1107/S1600536811038906/tk2794Isup2.hkl

Additional supplementary materials:  crystallographic information; 3D view; checkCIF report

## Figures and Tables

**Table:** 

Pt1—N1	2.003 (3)
Pt1—N3	2.014 (4)
Pt1—Cl2	2.2916 (15)
Pt1—Cl1	2.2918 (15)

**Table:** 

N1—Pt1—N3	80.25 (13)
Cl2—Pt1—Cl1	88.97 (5)

**Table 2:** Hydrogen-bond geometry (Å, °)

*D*—H⋯*A*	*D*—H	H⋯*A*	*D*⋯*A*	*D*—H⋯*A*
C3—H3⋯N2^i^	0.95	2.58	3.410 (6)	147
C4—H4⋯Cl1	0.95	2.57	3.180 (4)	123
C6—H6⋯Cl1^ii^	0.95	2.82	3.477 (5)	127
C6—H6⋯N4	0.95	2.58	3.056 (6)	111
C9—H9⋯Cl2	0.95	2.66	3.261 (5)	122
C13—H13⋯N4^iii^	0.95	2.61	3.468 (6)	151

Symmetry codes: (i) 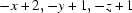; (ii) 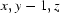; (iii) 
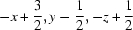
.

**Fig. 1.**
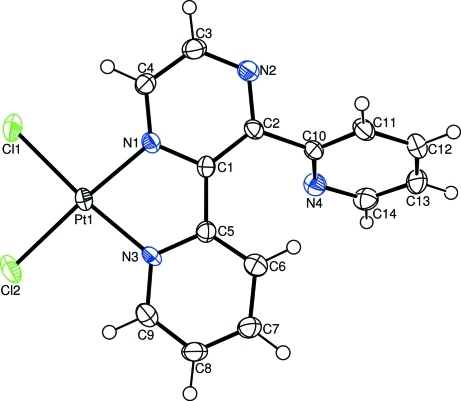
The structure of the title complex, with displacement ellipsoids drawn at the 50% probability level; H atoms are shown as small circles of arbitrary radius.

**Fig. 2.**
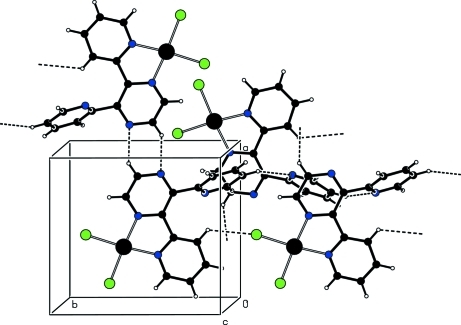
View of the unit-cell contents of the title complex. Intermolecular hydrogen-bond interactions are drawn with dashed lines.

## supplementary crystallographic information

### Comment

The title complex, [PtCl_2_(dpp)] (where dpp is 2,3-di-2-pyridylpyrazine, C_14_H_10_N_4_), is isomorphous with the analogous Pt^II^ complex [PtBr_2_(dpp)] (Ha, 2011).

In the complex, the Pt^II^ ion is four-coordinated in a distorted square-planar environment by two N atoms from the pyrazine ring and the one pyridyl ring of the chelating dpp ligand and two chloride anions (Fig. 1). The main contribution to the distortion of the square-plane is the tight N1—Pt1—N3 chelate angle of 80.25 (13)°, which results in slightly bent *trans* axes [<Cl1—Pt1—N3 = 174.63 (10)° and <Cl2—Pt1—N1 = 176.21 (10)°]. The pairs of Pt—N and Pt—Br bond lengths are experimentally equivalent (Table 1). In the molecule, the pyridyl ring coordinated to the Pt atom is inclined slightly to its carrier pyrazine ring, making dihedral angle of 13.5 (1)°. On the contrary, the uncoordinated pyridyl ring is inclined considerably to the pyrazine ring with a dihedral angle of 54.3 (2)°. The dihedral angle between the two pyridyl rings is 59.2 (2)°.

The complex molecules are assembled through intermolecular C—H···N and C—H···Cl hydrogen bonds to form a three-dimensional network (Fig. 2 and Table 2). There are also intramolecular C—H···N and C—H···Cl hydrogen bonds (Table 2). The complexes stack in columns along the *c* axis and display several intermolecular π-π interactions between the six-membered rings, with a shortest ring centroid-centroid distance of 4.213 (3) Å.

### Experimental

The title complex was obtained as a by-product from the reaction of K_2_PtCl_4_ (0.2077 g, 0.500 mmol) with 2,3-di-2-pyridylpyrazine (0.1173 g, 0.501 mmol) in MeOH (30 ml) and H_2_O (20 ml). After stirring of the reaction mixture for 48 h at room temperature, the formed precipitate was separated by filtration, washed with H_2_O and acetone, to give the main product as a red-brown powder (0.1323 g). The yellow by-product (0.0082 g) was obtained from the mixture of filtrate and washing solution. Crystals were obtained by slow evaporation from a CH_3_NO_2_ solution of the by-product.

### Refinement

H atoms were positioned geometrically and allowed to ride on their respective parent atoms [C—H = 0.95 Å and *U*_iso_(H) = 1.2*U*_eq_(C)]. The highest peak (1.83 e Å^-3^) and the deepest hole (-0.83 e Å^-3^) in the final difference Fourier map were located 0.89 Å and 0.88 Å from the Cl1 and Pt1 atoms, respectively.

### Crystal data

**Table:**

[PtCl_2_(C_14_H_10_N_4_)]	*F*(000) = 936
*M**_r_* = 500.25	*D*_x_ = 2.344 Mg m^−^^3^
Monoclinic, *P*2_1_/*n*	Mo *K*α radiation, λ = 0.71073 Å
Hall symbol: -P 2yn	Cell parameters from 5943 reflections
*a* = 8.894 (5) Å	θ = 2.4–28.3°
*b* = 9.711 (5) Å	µ = 10.27 mm^−^^1^
*c* = 16.461 (9) Å	*T* = 200 K
β = 94.429 (11)°	Block, yellow
*V* = 1417.5 (13) Å^3^	0.28 × 0.15 × 0.11 mm
*Z* = 4	

### Data collection

**Table:**

Bruker SMART 1000 CCD diffractometer	3370 independent reflections
Radiation source: fine-focus sealed tube	2756 reflections with *I* > 2σ(*I*)
graphite	*R*_int_ = 0.029
φ and ω scans	θ_max_ = 28.4°, θ_min_ = 2.4°
Absorption correction: multi-scan (*SADABS*; Bruker, 2000)	*h* = −11→11
*T*_min_ = 0.638, *T*_max_ = 1.000	*k* = −12→12
9749 measured reflections	*l* = −21→17

### Refinement

**Table:**

Refinement on *F*^2^	Primary atom site location: structure-invariant direct methods
Least-squares matrix: full	Secondary atom site location: difference Fourier map
*R*[*F*^2^ > 2σ(*F*^2^)] = 0.024	Hydrogen site location: inferred from neighbouring sites
*wR*(*F*^2^) = 0.056	H-atom parameters constrained
*S* = 1.02	*w* = 1/[σ^2^(*F*_o_^2^) + (0.0244*P*)^2^] where *P* = (*F*_o_^2^ + 2*F*_c_^2^)/3
3370 reflections	(Δ/σ)_max_ = 0.001
190 parameters	Δρ_max_ = 1.83 e Å^−^^3^
0 restraints	Δρ_min_ = −0.83 e Å^−^^3^

### Special details

**Table:**

Geometry. All e.s.d.'s (except the e.s.d. in the dihedral angle between two l.s. planes) are estimated using the full covariance matrix. The cell e.s.d.'s are taken into account individually in the estimation of e.s.d.'s in distances, angles and torsion angles; correlations between e.s.d.'s in cell parameters are only used when they are defined by crystal symmetry. An approximate (isotropic) treatment of cell e.s.d.'s is used for estimating e.s.d.'s involving l.s. planes.
Refinement. Refinement of *F*^2^ against ALL reflections. The weighted *R*-factor *wR* and goodness of fit *S* are based on *F*^2^, conventional *R*-factors *R* are based on *F*, with *F* set to zero for negative *F*^2^. The threshold expression of *F*^2^ > σ(*F*^2^) is used only for calculating *R*-factors(gt) *etc*. and is not relevant to the choice of reflections for refinement. *R*-factors based on *F*^2^ are statistically about twice as large as those based on *F*, and *R*- factors based on ALL data will be even larger.

### Fractional atomic coordinates and isotropic or equivalent isotropic displacement parameters (Å^2^)

**Table:**

	*x*	*y*	*z*	*U*_iso_*/*U*_eq_	
Pt1	0.364118 (17)	0.639554 (16)	0.379000 (10)	0.02301 (6)	
Cl1	0.44494 (14)	0.85790 (11)	0.41230 (9)	0.0410 (3)	
Cl2	0.12714 (12)	0.72595 (12)	0.34708 (7)	0.0362 (3)	
N1	0.5650 (4)	0.5518 (3)	0.4076 (2)	0.0215 (7)	
N2	0.8366 (4)	0.4154 (4)	0.4387 (2)	0.0260 (8)	
N3	0.3108 (4)	0.4412 (4)	0.3556 (2)	0.0229 (8)	
N4	0.6997 (4)	0.1724 (4)	0.3059 (2)	0.0288 (8)	
C1	0.5721 (4)	0.4119 (4)	0.3971 (2)	0.0207 (8)	
C2	0.7128 (4)	0.3476 (4)	0.4088 (2)	0.0209 (9)	
C3	0.8227 (5)	0.5502 (4)	0.4532 (3)	0.0290 (10)	
H3	0.9075	0.5992	0.4769	0.035*	
C4	0.6902 (5)	0.6200 (4)	0.4350 (3)	0.0259 (9)	
H4	0.6870	0.7171	0.4418	0.031*	
C5	0.4224 (5)	0.3480 (4)	0.3745 (3)	0.0228 (9)	
C6	0.3948 (5)	0.2085 (5)	0.3771 (3)	0.0297 (10)	
H6	0.4729	0.1454	0.3936	0.036*	
C7	0.2471 (5)	0.1621 (4)	0.3545 (3)	0.0300 (10)	
H7	0.2236	0.0668	0.3565	0.036*	
C8	0.1382 (5)	0.2558 (5)	0.3297 (3)	0.0324 (11)	
H8	0.0400	0.2257	0.3108	0.039*	
C9	0.1721 (5)	0.3938 (5)	0.3324 (3)	0.0314 (10)	
H9	0.0945	0.4582	0.3173	0.038*	
C10	0.7422 (4)	0.2020 (4)	0.3837 (2)	0.0216 (9)	
C11	0.8186 (5)	0.1104 (4)	0.4368 (3)	0.0280 (10)	
H11	0.8450	0.1352	0.4919	0.034*	
C12	0.8553 (5)	−0.0175 (5)	0.4078 (3)	0.0349 (11)	
H12	0.9090	−0.0819	0.4424	0.042*	
C13	0.8132 (5)	−0.0508 (5)	0.3275 (3)	0.0346 (11)	
H13	0.8371	−0.1380	0.3059	0.041*	
C14	0.7356 (5)	0.0462 (5)	0.2800 (3)	0.0320 (11)	
H14	0.7053	0.0223	0.2251	0.038*	

### Atomic displacement parameters (Å^2^)

**Table:**

	*U*^11^	*U*^22^	*U*^33^	*U*^12^	*U*^13^	*U*^23^
Pt1	0.02249 (10)	0.02038 (10)	0.02625 (10)	0.00622 (7)	0.00249 (7)	0.00141 (7)
Cl1	0.0379 (7)	0.0187 (6)	0.0665 (9)	0.0050 (5)	0.0044 (6)	−0.0015 (5)
Cl2	0.0281 (6)	0.0377 (6)	0.0421 (7)	0.0163 (5)	−0.0008 (5)	0.0003 (5)
N1	0.0188 (17)	0.0216 (18)	0.0243 (18)	0.0027 (14)	0.0033 (14)	0.0017 (14)
N2	0.0217 (18)	0.0280 (19)	0.028 (2)	0.0017 (16)	−0.0002 (15)	−0.0016 (16)
N3	0.0141 (16)	0.026 (2)	0.0280 (19)	0.0044 (15)	−0.0008 (14)	0.0001 (15)
N4	0.028 (2)	0.031 (2)	0.028 (2)	0.0018 (16)	0.0037 (16)	−0.0023 (16)
C1	0.024 (2)	0.018 (2)	0.021 (2)	0.0021 (17)	0.0024 (17)	0.0021 (16)
C2	0.019 (2)	0.023 (2)	0.021 (2)	0.0014 (16)	−0.0006 (16)	0.0040 (16)
C3	0.027 (2)	0.030 (2)	0.030 (2)	−0.0040 (19)	−0.0012 (19)	−0.0042 (19)
C4	0.024 (2)	0.022 (2)	0.032 (2)	−0.0016 (17)	0.0037 (19)	−0.0034 (17)
C5	0.023 (2)	0.021 (2)	0.025 (2)	0.0007 (17)	0.0037 (17)	0.0001 (17)
C6	0.024 (2)	0.031 (2)	0.035 (3)	0.001 (2)	0.004 (2)	0.000 (2)
C7	0.024 (2)	0.028 (3)	0.039 (3)	−0.0041 (19)	0.006 (2)	−0.0041 (19)
C8	0.021 (2)	0.034 (3)	0.042 (3)	−0.0058 (19)	0.002 (2)	−0.009 (2)
C9	0.023 (2)	0.033 (3)	0.038 (3)	0.0064 (19)	0.000 (2)	−0.001 (2)
C10	0.0161 (19)	0.023 (2)	0.026 (2)	0.0019 (17)	0.0046 (17)	−0.0011 (17)
C11	0.024 (2)	0.031 (3)	0.028 (2)	0.0054 (18)	−0.0043 (19)	−0.0010 (18)
C12	0.031 (3)	0.028 (2)	0.045 (3)	0.008 (2)	−0.006 (2)	0.001 (2)
C13	0.032 (3)	0.025 (2)	0.048 (3)	0.005 (2)	0.006 (2)	−0.008 (2)
C14	0.032 (2)	0.035 (3)	0.029 (3)	−0.002 (2)	0.002 (2)	−0.008 (2)

### Geometric parameters (Å, °)

**Table:**

Pt1—N1	2.003 (3)	C4—H4	0.9500
Pt1—N3	2.014 (4)	C5—C6	1.378 (6)
Pt1—Cl2	2.2916 (15)	C6—C7	1.411 (6)
Pt1—Cl1	2.2918 (15)	C6—H6	0.9500
N1—C4	1.344 (5)	C7—C8	1.368 (6)
N1—C1	1.372 (5)	C7—H7	0.9500
N2—C3	1.337 (5)	C8—C9	1.374 (6)
N2—C2	1.343 (5)	C8—H8	0.9500
N3—C9	1.344 (5)	C9—H9	0.9500
N3—C5	1.361 (5)	C10—C11	1.388 (6)
N4—C10	1.339 (5)	C11—C12	1.379 (6)
N4—C14	1.344 (5)	C11—H11	0.9500
C1—C2	1.399 (5)	C12—C13	1.383 (7)
C1—C5	1.489 (6)	C12—H12	0.9500
C2—C10	1.501 (5)	C13—C14	1.375 (6)
C3—C4	1.373 (6)	C13—H13	0.9500
C3—H3	0.9500	C14—H14	0.9500
			
N1—Pt1—N3	80.25 (13)	C6—C5—C1	124.0 (4)
N1—Pt1—Cl2	176.21 (10)	C5—C6—C7	118.0 (4)
N3—Pt1—Cl2	96.16 (10)	C5—C6—H6	121.0
N1—Pt1—Cl1	94.59 (10)	C7—C6—H6	121.0
N3—Pt1—Cl1	174.63 (10)	C8—C7—C6	119.3 (4)
Cl2—Pt1—Cl1	88.97 (5)	C8—C7—H7	120.3
C4—N1—C1	119.1 (3)	C6—C7—H7	120.3
C4—N1—Pt1	124.8 (3)	C7—C8—C9	119.3 (4)
C1—N1—Pt1	116.2 (3)	C7—C8—H8	120.3
C3—N2—C2	117.5 (4)	C9—C8—H8	120.3
C9—N3—C5	118.3 (4)	N3—C9—C8	122.5 (4)
C9—N3—Pt1	125.4 (3)	N3—C9—H9	118.7
C5—N3—Pt1	115.8 (3)	C8—C9—H9	118.7
C10—N4—C14	116.3 (4)	N4—C10—C11	123.6 (4)
N1—C1—C2	118.3 (4)	N4—C10—C2	115.0 (4)
N1—C1—C5	113.3 (3)	C11—C10—C2	121.1 (4)
C2—C1—C5	128.4 (4)	C12—C11—C10	118.4 (4)
N2—C2—C1	122.1 (4)	C12—C11—H11	120.8
N2—C2—C10	114.1 (3)	C10—C11—H11	120.8
C1—C2—C10	123.7 (4)	C11—C12—C13	119.2 (4)
N2—C3—C4	122.3 (4)	C11—C12—H12	120.4
N2—C3—H3	118.9	C13—C12—H12	120.4
C4—C3—H3	118.9	C14—C13—C12	118.1 (4)
N1—C4—C3	120.3 (4)	C14—C13—H13	121.0
N1—C4—H4	119.8	C12—C13—H13	121.0
C3—C4—H4	119.8	N4—C14—C13	124.3 (4)
N3—C5—C6	122.1 (4)	N4—C14—H14	117.8
N3—C5—C1	113.7 (3)	C13—C14—H14	117.8
			
N3—Pt1—N1—C4	−179.3 (3)	Pt1—N3—C5—C1	−9.1 (4)
Cl1—Pt1—N1—C4	−0.8 (3)	N1—C1—C5—N3	10.1 (5)
N3—Pt1—N1—C1	1.3 (3)	C2—C1—C5—N3	−170.4 (4)
Cl1—Pt1—N1—C1	179.8 (3)	N1—C1—C5—C6	−166.0 (4)
N1—Pt1—N3—C9	176.5 (4)	C2—C1—C5—C6	13.5 (7)
Cl2—Pt1—N3—C9	−2.3 (3)	N3—C5—C6—C7	3.8 (6)
N1—Pt1—N3—C5	4.6 (3)	C1—C5—C6—C7	179.6 (4)
Cl2—Pt1—N3—C5	−174.2 (3)	C5—C6—C7—C8	1.2 (6)
C4—N1—C1—C2	−5.3 (5)	C6—C7—C8—C9	−4.3 (7)
Pt1—N1—C1—C2	174.0 (3)	C5—N3—C9—C8	2.2 (6)
C4—N1—C1—C5	174.2 (3)	Pt1—N3—C9—C8	−169.5 (3)
Pt1—N1—C1—C5	−6.4 (4)	C7—C8—C9—N3	2.7 (7)
C3—N2—C2—C1	−3.5 (6)	C14—N4—C10—C11	−0.2 (6)
C3—N2—C2—C10	172.2 (3)	C14—N4—C10—C2	174.8 (3)
N1—C1—C2—N2	7.7 (6)	N2—C2—C10—N4	−122.6 (4)
C5—C1—C2—N2	−171.8 (4)	C1—C2—C10—N4	52.9 (5)
N1—C1—C2—C10	−167.5 (4)	N2—C2—C10—C11	52.5 (5)
C5—C1—C2—C10	13.0 (6)	C1—C2—C10—C11	−131.9 (4)
C2—N2—C3—C4	−3.1 (6)	N4—C10—C11—C12	1.1 (6)
C1—N1—C4—C3	−0.9 (6)	C2—C10—C11—C12	−173.6 (4)
Pt1—N1—C4—C3	179.8 (3)	C10—C11—C12—C13	−0.9 (7)
N2—C3—C4—N1	5.4 (6)	C11—C12—C13—C14	−0.1 (7)
C9—N3—C5—C6	−5.5 (6)	C10—N4—C14—C13	−0.9 (6)
Pt1—N3—C5—C6	167.0 (3)	C12—C13—C14—N4	1.1 (7)
C9—N3—C5—C1	178.3 (3)		

### Hydrogen-bond geometry (Å, °)

**Table:**

*D*—H···*A*	*D*—H	H···*A*	*D*···*A*	*D*—H···*A*
C3—H3···N2^i^	0.95	2.58	3.410 (6)	147
C4—H4···Cl1	0.95	2.57	3.180 (4)	123
C6—H6···Cl1^ii^	0.95	2.82	3.477 (5)	127
C6—H6···N4	0.95	2.58	3.056 (6)	111
C9—H9···Cl2	0.95	2.66	3.261 (5)	122
C13—H13···N4^iii^	0.95	2.61	3.468 (6)	151

Symmetry codes: (i) −*x*+2, −*y*+1, −*z*+1; (ii) *x*, *y*−1, *z*; (iii) −*x*+3/2, *y*−1/2, −*z*+1/2.
